# Limited discriminatory value of C-reactive protein for viral coinfection and respiratory severity in children evaluated in the emergency department for acute respiratory tract illness

**DOI:** 10.3389/fped.2026.1854804

**Published:** 2026-07-22

**Authors:** Rashed A. Hasan, Brian M. Nolan

**Affiliations:** 1 Department of Pediatrics, Hurley Medical Center, Flint, MI, United States; 2 Department of Pediatrics, Michigan State University, East Lansing, MI, United States

**Keywords:** bronchiolitis, C-reactive protein, respiratory syncytial virus, rhinovirus, viral coinfection

## Abstract

**Background:**

C-reactive protein is often measured in children who present to an acute care facility with acute respiratory tract illness (ARTI), and multiplex polymerase chain reaction (PCR) testing on respiratory samples frequently identifies more than one pathogen per encounter in children with ARTI. Whether CRP differentiates single-virus from multiple virus infections or improves prediction of clinical course of ARTI is uncertain.

**Objective:**

To assess whether age-stratified CRP differs by viral coinfection status, and whether CRP adds discriminatory value beyond clinical variables for predicting advanced respiratory support, after accounting for selection bias, probable bacterial coinfection, repeated encounters and overadjustment.

**Methods:**

This was a retrospective encounter-level cohort study of 17,089 encounters with ARTI in which multiplex respiratory polymerase chain reaction (PCR) testing was performed in the emergency department (ED) between Jan 1, 2019, and Dec 31, 2025. Encounters were not restricted to pathogen-positive results on the PCR. The analytic cohort comprised encounters with a measured CRP (*n* = 4,946) drawn from 17,089 total ED encounters. We compared CRP-tested vs. untested encounters using standardized mean differences. Single-virus and multi-virus encounters were compared overall, by virus, by age stratum (< 6months, 6–23 months, 2–5 years, 6–12 years, and 13–17 years.) and by hospital disposition. Sensitivity analyses (a) restricted to encounters without probable bacterial coinfection (no sepsis, pneumonia, or antibiotics use), (b) restricted to one encounter per patient, and (c) re-estimated logistic models without potential mediators. Multivariable logistic regression for advanced respiratory support used a parsimonious specification (age, sex, prematurity, initial SpO2, CRP, virus features); a model including severity-of-illness was reported only as a sensitivity exhibit because severity is partly defined by outcome. Discrimination was summarized by AUC with bootstraps 95% CIs, and CRP thresholds were evaluated for sensitivity, specificity, and positive likelihood ratio (LR). The Benjamini-Hochberg FDR was used to control for multiple testing.

**Results:**

Compared with untested encounters, tested patients were sicker (pediatric ward admission 74.0% vs. 36.3% [SMD 0.82]; pneumonia 23.4% vs. 9.6% [SMD 0.38]; antibiotic use 59.5% vs. 20.9% [SMD 0.86%]; PICU admission 45.1% vs. 28.5% [SMD 0.35]. RV/EV positivity was lower among tested encounters (30.7% vs. 41.4%, SMD – 0.23]. Within the CRP cohort, 3,037 (61.4%) met criteria for probably bacterial coinfection. After excluding probably bacterial coinfection, only RSV retained as significant single vs. coinfection CRP difference (3.0 vs. 5.2 mg/L, *q* = 0.005). Significant age x coinfection interactions on log-CRP were detected for RSV, common coronaviruses, adenovirus, and SARS-Cov-2 (all *q*, 0.05). The coinfection-CRP signal was concentrated in ED-only patients (*p* = 0.008) and was absent among pediatric ward (*p* = 0.30) or PICU admissions (*p* = 0.58). CRP alone did not show discrimination for advanced respiratory support (AU 0.483); a clinical model achieved AUC 0.75, with no improvement after CRP (AUC 0.75). The CRP per-10-mg/L coefficient had aOR 1.013 (1.001–1.026, *p* = 0.036) in the parsimonious model and aOR 1.000 (0.983–1.018, *p* = 0.96) when restricted to one-encounter-per-patient.

**Conclusions:**

CRP testing is preferentially measured in patients who already appear sicker. After accounting for probably bacterial infection, CRP does not reliably distinguish single from multi-virus ARTI except for RSV in younger children, and it does not provide clinically useful incremental discrimination beyond simple clinical variables for advanced respiratory support.

## Introduction

C-reactive protein (CRP) is an acute phase reactant synthesized by hepatocytes in response to pro-inflammatory cytokines, and is widely used as a marker of systemic inflammation, particularly in infection (1, 2). In children with clinically significant acute respiratory tract infection (ARTI) that manifests as dyspnea, tachypnea, hypoxemia or hypercarbia, serum CRP concentration is frequently measured to support decisions about hospitalization, antibiotic utilization and escalation of respiratory support (3). However, interpretation of CRP concentration in the setting of viral ARTI is subject to a number of methodological flaws that clinicians need to pay attention to. Clinicians are more likely to request measurement of CRP concentration in children who appear more severely ill or in patients in whom associated bacterial infections are suspected, introducing substantial selection bias. Multivariable models that evaluate for escalation of respiratory support measures and adjust for illness severity can have flaws because severity of illness itself is part of the escalation of respiratory support (4, 5).

Multiplex respiratory polymerase chain reaction (PCR) testing has expanded the etiologic characterization of pediatric ARTI, but it has also introduced complexity in the clinical interpretation of results, since two or more viruses are commonly detected during a single encounter (6). Whether viral coinfection systematically changes the inflammatory response that can be distinguished by measurement of CRP is unclear (7–10). Additionally, surrogate markers of bacterial superinfection including radiographic pneumonia, clinical features of sepsis and antibiotic utilization are common in the subset of CRP-tested children with viral ARTI and pooling these patients with viral-only encounters can mask or mislead the overall clinical effects. And finally, retrospective cohorts often include repeated encounters per child, treating each encounter as independent can inflate apparent CRP effects particularly with viruses associated with prolonged shedding (6, 7).

In this study, we aimed to investigate the relationship between CRP, viral coinfection, and respiratory severity in a retrospective cohort of children evaluated in the ED for ARTI, using a study design tailored to explicitly address each of the above methodological issues.

The primary objective of this study was to test whether multi-virus encounters differ in CRP concentration from single-virus encounters overall and within age strata.

Secondary objectives included:

- To Characterize the CRP-tested versus untested populations to quantify selection bias.
- To repeat all coinfection comparisons after excluding encounters with probable bacterial coinfection
- To evaluate the discriminatory value of CRP for advanced respiratory support measures relative to a parsimonious clinical model
- To assess for robustness of repeated encounters and for overadjustment.

Our hypothesis was that any association of CRP with viral coinfection or short-term respiratory severity would be modest, age- and pathogen-specific, and largely attributable to bacterial coinfection rather than to viral coinfection *per se*.

## Methods

### Study design and setting

We conducted a retrospective cohort study of children evaluated in the ED for ARTI at a tertiary care center between January 1, 2019, and December 31, 2025. Eligibility criteria included children < 18 years of age who were evaluated in the ED for clinically significant ARTI and underwent respiratory PCR testing. Clinically significant ARTI was defined as one or more of the following: respiratory distress with tachypnea for age, retractions, hypoxemia (SpO_2_) < 90% while breathing ambient air, or hypercarbia (PCO_2_) > 45 torr on blood gas analysis.

### Participants and cohort definitions

The overall cohort included 17,089 encounters in which multiplex respiratory PCR testing (Biofire Respiratory panel 2.1, BioMerieux, Salt Lake city, UT, USA) was performed in the ED between Jan 1, 2019, and Dec 31, 2025. Encounters were not restricted to pathogen-positive results on the PCR. The CRP cohort comprised the subset with at least one PCR (Atelica Ch analyzer, SIemmens Healthneers, Melvin, PA, USA) measured at the time of presentation to the ED during the same encounter. Single-virus encounters were defined as encounters with exactly one positive virus on the multiplex PCR panel; multi-virus (coinfection) encounters were defined as two or more positive viruses identified on the respiratory multiplex PCR test.

### Variables and definitions

Demographics, clinical data including history of preterm birth, initial vital signs including first heart rate, first respiratory rate, first temperature, first blood pressure and pulse oximetry (SpO_2_), disposition, respiratory support measures used to include high-flow nasal cannula (HFNC), CPAP/BPAP, invasive mechanical ventilation were extracted from electronic health records. Pneumonia was diagnosed radiographically as the presence of infiltrates or opacification on a plain chest radiograph, involving one or more lobes, as reported by an independent radiologist. Sepsis was defined according to International standards (11).

The primary exposure was viral coinfection status (single virus vs. multi-virus). The primary biomarker was the first CRP measured during the encounter in the ED; values reported as inequities were assigned the numeric bound. Pathogen indicators were obtained from the multiplex PCR panel for rhinovirus and/or enterovirus (RV/EV), RSV, influenza A/B, adenovirus, Human metapneumovirus (hMPV), common human coronaviruses, SARS-Cov-2, and parainfluenza 1–4, Mycoplasma pneumoniae, chlamydia and Bordetella pertussis.

Age was analyzed continuously and grouped into < 6 months, 6–23 months 2–5 years, 6–12 years, and older than years. The primary clinical outcome was advanced respiratory support during the encounter, defined as receipt of high-flow nasal cannula (HFNC), CPAP/BPAP, or invasive mechanical ventilation. Probable bacterial coinfection was defined *a priori* as a clinical diagnosis of pneumonia (defined radiographically as the presence of infiltrates or opacifications involving one or more lobes on a plain frontal chest radiograph, as reported independently by a radiologist), sepsis using the international definition of sepsis criteria (11), or receipt of an antibiotic in the ED during the encounter; this composite was used as surrogate exposure to distinguish plausibly viral-only encounters from those with bacterial involvement.

### Statistical analysis

Continuous data were summarized using median and interquartile ranges and were analyzed using the Mann–Whitney U test, and categorical data presented as counts (%) and were analyzed using chi-square analysis.

We characterized selection bias between tested and untested encounters using SMDs in addition to *p*-values, with SMD > 0.10 considered indicative of imbalance. We compared single virus vs. coinfection within each pathogen subgroup, both in the full CRP cohort and in a viral-only sensitivity cohort excluding probable bacterial coinfection. Age-stratified differences were evaluated within RSV-positive encounters and via pathogen-by-age interaction tests on log_10_ [CRP] using OLS with a likelihood-ratio *F*-test for the interaction term.

Multivariable logistic regression was used to estimate the association of CRP with advanced respiratory support. The parsimonious primary model (named Model C) included age, sex, preterm birth, initial SpO_2_, CRP (per 10 mg/L), multi-virus status, and RV/EV status.

Illness severity was not included in the primary model because it is partly defined by respiratory support measures and would introduce overadjustment, however we have included, a model including severity (named Model D) as a sensitivity exhibit only.

We used variance inflation factors to evaluate multicollinearity.

Discrimination was summarized by AUC with 1,000-resample bootstrap 95% CIs. Pre-specified CRP thresholds (≥ 10, ≥ 20, ≥ 40, ≥ 80, ≥ 100 mg/L) were evaluated for sensitivity, specificity, PPV, NPV, and positive likelihood ratio.

To address repeated encounters, all primary analyses were repeated using the first encounter per unique patient. Multiple comparisons were controlled with the Benjamini-Hochberg FDR within each family of tests.

Two-sided *p* values < 0.05 and FDR *q*-values < 0.05 were considered statistically significant. Statistical analyses were completed using SPSS V 31 (IBM Corporation, Armonk, NY, USA) and R version 4.2.2 (Posit PBC, Boston, MA, USA).

### Ethics

The instituational review board approved the study and waived the need for a consent. The study participants were treated in accordance with ethical principles of conducting research of the declaration of Helsinki of 1975 and as revised in 1983.

## Results

### Cohort description and selection bias

Between January 1, 2019, and December 31, 2025, 17,089 ED encounters met inclusion criteria. Table 1 shows the characteristics of the cohort. CRP was measured in 4,946 (28.9%), which comprise the analytic cohort and Table 2 presents a comparison of the two groups.

**Table 1:** Demographic, clinical and microbiological characteristics of the total cohort (2019–2025).

Characteristic	Total encounters (*N* = 17,089)
Age, months	26.0 (9.0–71.0)
Female sex	7,777 (45.5%)
Preterm birth	2,478 (14.5%)
ANY positive pathogen identified	12,405 (72.6%)
RV/EV	6,550 (38.3%)
Adenovirus	1,584 (9.3%)
Influenza	1,010 (5.9%)
Parainfluenza virus 1	290 (1.7%)
Parainfluenza virus 2	242 (1.4%)
Parainfluenza virus 3	613 (3.6%)
Parainfluenza virus 4	194 (1.1%)
Human metapneumovirus	640 (3.7%)
Mycoplasma pneumoniae	302 (1.8%)
Chlamydia pneumoniae	11 (0.1%)
Bordetella	50 (0.3%)
Common human coronaviruses	876 (5.1%)
RSV	2,523 (14.8%)
SARS-CoV-2 (any year, 2020–2024)	684 (4.0%)
Pediatric floor admission	8,072 (47.2%)
PICU admission	5,693 (33.3%)
Pneumonia	2,322 (13.6%)
Sepsis	375 (2.2%)
Antibiotics administered	5,478 (32.1%)
Hospital length of stay, days	1.00 (0.00–3.00)
Discharged	14,751 (86.3%)
30-day readmission	2,323 (13.6%)
Death	15 (0.1%)

Continuous data are presented as median with interquartile and categorical data are presented as counts (%). There were no encounters with SARS-Cov-2 in 2025.

**Table 2:** Comparison of the cohort with and without C-reactive protein measurement (2019–2025).

Characteristic	CRP measured (*N* = 4,946)	No CRP (*N* = 12,143)	*p*-value
Age, months	30.0 (9.0–83.0)	25.0 (10.0–68.0)	<0.001
Female sex	2,234 (45.2%)	5,543 (45.6%)	0.568
Preterm birth	778 (15.7%)	1,700 (14.0%)	0.004
ANY positive pathogen identified	3,036 (61.4%)	9,369 (77.2%)	<0.001
RV/EV	1,519 (30.7%)	5,031 (41.4%)	<0.001
Adenovirus	400 (8.1%)	1,184 (9.8%)	<0.001
Influenza	255 (5.2%)	755 (6.2%)	0.008
Parainfluenza virus 1	56 (1.1%)	234 (1.9%)	<0.001
Parainfluenza virus 2	47 (1.0%)	195 (1.6%)	0.001
Parainfluenza virus 3	130 (2.6%)	483 (4.0%)	<0.001
Parainfluenza virus 4	32 (0.6%)	162 (1.3%)	<0.001
Human metapneumovirus	189 (3.8%)	451 (3.7%)	0.738
Mycoplasma pneumoniae	80 (1.6%)	222 (1.8%)	0.343
Chlamydia pneumoniae	2 (0.0%)	9 (0.1%)	0.740
Bordetella	12 (0.2%)	38 (0.3%)	0.440
Common human coronaviruses	185 (3.7%)	691 (5.7%)	<0.001
RSV	689 (13.9%)	1,834 (15.1%)	0.050
SARS-CoV-2 (any year, 2020–2024)	173 (3.5%)	511 (4.2%)	0.032
Pediatric floor admission	3,660 (74.0%)	4,412 (36.3%)	<0.001
PICU admission	2,231 (45.1%)	3,462 (28.5%)	<0.001
Pneumonia	1,158 (23.4%)	1,164 (9.6%)	<0.001
Sepsis	295 (6.0%)	80 (0.7%)	<0.001
Antibiotics administered	2,941 (59.5%)	2,537 (20.9%)	<0.001
Hospital length of stay, days	3.0 (1.0–4.0)	1.0 (0.0–2.0)	<0.001
Discharged	4,275 (86.4%)	10,476 (86.3%)	0.780
30-day readmission	666 (13.5%)	1,657 (13.6%)	0.755
Death	5 (0.1%)	10 (0.1%)	0.708

Continuous data are presented as median with interquartile and categorical data are presented as counts (%). *P* < 0.05 was considered statistically significant.

The median age in the CRP cohort was 30 months (IQR 9–83), and RV/EV was the most common virus and was identified in 1,519 (30.7%) and any-virus coinfection was observed in 1.796 (36.3%). CRP-tested encounters differed substantially from untested encounters across markers of acuity (Table 3). An antibiotic (usually ceftriaxone intravenously) was been administered in the ED in 59.5% of CRP tested vs. 20.9% of untested encounters (SMD 0.86), pediatric ward care noted in 74.0% vs. 36.3% (SMD 0.82), radiographic pneumonia diagnosed in 23.4% vs. 9.6% (SMD 0.38), oxygen therapy was provided in 43.6% vs. 26.3% (SMD 0.37), and PICU-level care occurred in 45.1% vs. 28.5% (SMD 0.35).

**Table 3:** Comparison of CRP-tested and untested encounters (*N* = 17,089).

Variable	CRP tested (*n* = 4,946)	CRP not tested (*n* = 12,143)	*p*-value	SMD
Age (months)	30.0 [9.0–83.0]	25.0 [10.0–68.0]	0.0006	0.12
RV/EV positive	1,519 (30.7%)	5,031 (41.4%)	<0.001	−0.225
Coinfection identified	1,796 (36.3%)	5,445 (44.8%)	<0.001	−0.174
Adenovirus	400 (8.1%)	1,184 (9.8%)	<0.001	−0.058
RSV	689 (13.9%)	1,834 (15.1%)	0.0528	−0.033
Influenza	255 (5.2%)	755 (6.2%)	0.0084	−0.046
hMPV	189 (3.8%)	451 (3.7%)	0.7716	0.006
Coronavirus (non-SARS)	185 (3.7%)	691 (5.7%)	<0.001	−0.092
SARS-CoV-2	173 (3.5%)	511 (4.2%)	0.0352	−0.037
Antibiotics given	2,941 (59.5%)	2,537 (20.9%)	<0.001	0.856
Pneumonia diagnosis	1,158 (23.4%)	1,164 (9.6%)	<0.001	0.379
Sepsis	295 (6.0%)	80 (0.7%)	<0.001	0.3
Respiratory failure	1,594 (32.2%)	2,249 (18.5%)	< 0.001	0.319
Oxygen therapy	2,153 (43.6%)	3,161 (26.3%)	< 0.001	0.371
High flow	1,202 (100.0%)	1,730 (100.0%)	<0.001	0.0
CPAP/BPAP	774 (100.0%)	789 (100.0%)	<0.001	0.0
Mechanical ventilation	219 (100.0%)	171 (100.0%)	< 0.001	0.0
Hospitalized (Peds ward)	3,660 (74.0%)	4,412 (36.3%)	<0.001	0.818
PICU admission	2,231 (45.1%)	3,462 (28.5%)	<0.001	0.349
Preterm	778 (15.7%)	1,700 (14.0%)	<0.0039	0.049

SMD, standardized mean difference; SMD >0.10 indicates meaningful imbalance. Tested encounters are markedly enriched for hospitalization, antibiotic use, and indicators of severe illness.

In contrast, RV/EV positivity (30.7% vs. 41.4%, SMD – 0.23) and any-virus coinfection (36.3% vs. 44.8%, SMD -0.17) were less frequent among CRP-tested encounters.

### Probable bacterial coinfection in the CRP cohort

Within the CRP cohort, 3,037 encounters (61.4%) met the composite definition of probable bacterial coinfection (radiographic pneumonia, sepsis, or antibiotic administration in the ED). The remaining 1,909 encounters formed a viral-only sensitivity cohort.

### CRP differences by coinfection status: full vs. viral-only cohort

In analyses comparing single-virus detection with viral co-detection among CRP-tested encounters, RSV co-detection was associated with higher CRP than RSV alone in both the full CRP cohort and the viral-only cohort after false-discovery-rate correction. Parainfluenza co-detection was associated with higher CRP in the full CRP cohort but not after restricting to viral-only encounters. Other viruses did not show consistent differences after FDR correction (Table 4).

**Table 4:** CRP concentrations in single-virus versus multi-virus respiratory PCR detections Among CRP-tested encounters.

Cohort	Virus	Single *n*	Single CRP median [IQR]	Coinf *n*	Coinf CRP median [IQR]	*p*	*q* (FDR)
Full CRP cohort	Adenovirus	132	23.8 [8.3–56.3]	268	13.9 [5.8–34.6]	0.0227	0.0605
Full CRP cohort	RSV	429	7.4 [1.9–27.2]	260	10.9 [3.9–34.3]	0.0049	0.0196
Full CRP cohort	Influenza	185	7.5 [3.6–20.6]	70	10.8 [4.7–30.9]	0.2623	0.4197
Full CRP cohort	hMPV	130	19.7 [4.4–61.4]	59	11.4 [5.2–40.2]	0.4004	0.5339
Full CRP cohort	Coronavirus	81	10.0 [1.7–41.0]	104	8.2 [4.3–28.6]	0.674	0.7703
Full CRP cohort	SARS-CoV-2	126	6.9 [1.9–20.3]	47	5.0 [1.5–30.4]	0.9429	0.9429
Full CRP cohort	RV/EV	1,093	10.2 [3.1–32.6]	426	11.4 [4.3–36.2]	0.0485	0.097
Full CRP cohort	Parainfluenza	159	5.6 [0.9–21.7]	106	13.4 [3.7–34.0]	0.0008	0.0064
Viral-only (excl. probable bacterial)	Adenovirus	71	13.6 [4.7–39.8]	113	8.1 [3.4–19.4]	0.031	0.124
Viral-only (excl. probable bacterial)	RSV	154	3.0 [0.8–6.5]	95	5.2 [3.0–17.5]	0.0006	0.0048
Viral-only (excl. probable bacterial)	Influenza	53	5.0 [3.4–8.5]	9	11.4 [4.0–15.6]	0.1228	0.2456
Viral-only (excl. probable bacterial)	hMPV	34	5.9 [1.7–15.3]	12	6.6 [6.0–12.3]	0.3229	0.5166
Viral-only (excl. probable bacterial)	Coronavirus	38	6.9 [1.1–22.9]	39	6.3 [3.9–19.0]	0.7482	0.8551
Viral-only (excl. probable bacterial)	SARS-CoV-2	56	3.3 [0.9–7.7]	21	4.0 [1.0–9.5]	0.9863	0.9863
Viral-only (excl. probable bacterial)	RV/EV	527	6.6 [1.9–16.2]	168	6.3 [1.8–14.4]	0.7002	0.8551
Viral-only (excl. probable bacterial)	Parainfluenza	72	3.1 [0.6–11.6]	37	4.8 [2.3–19.4]	0.0608	0.1621

CRP values are shown as median [IQR] in mg/L among encounters with CRP testing. For each virus, encounters were classified as single-virus detection when that virus was the only respiratory virus identified and as multi-virus detection when that virus was detected with at least one additional respiratory virus. Analyses are shown for the full CRP cohort and for a viral-only cohort, which excluded encounters with radiographic pneumonia, sepsis, or antibiotic administration. Comparisons between single-virus and multi-virus groups used the Mann–Whitney U test. The q value represents the Benjamini-Hochberg false-discovery-rate adjusted *p* value within each cohort. Blank note fields indicate no additional note; strata with insufficient sample size were not tested.

### Age x virus interaction

**Figure 1:**
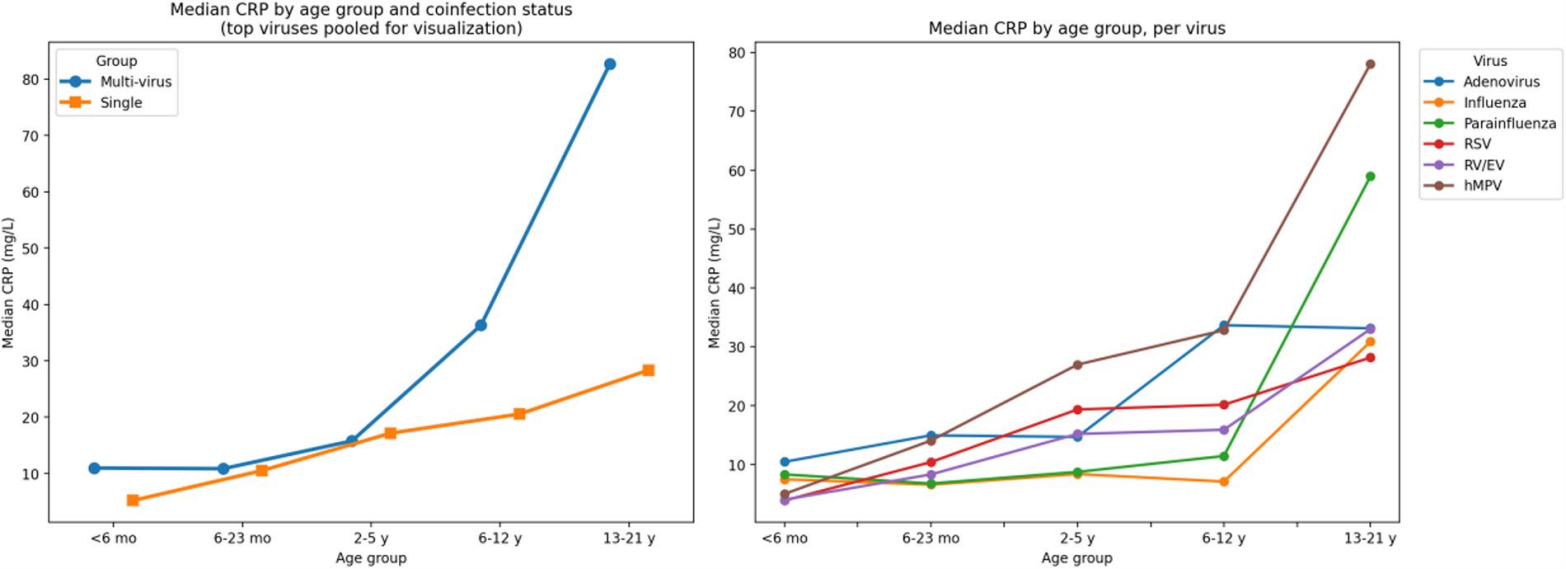
Median CRP by age group, stratified by virus and coinfection status. Left: median CRP by age group and coinfection status (single vs. multi-virus) for the most common viruses pooled. Right: trajectory of median CRP across age groups for each virus. Patterns are pathogen-specific and non-monotonic, illustrating significant age x virus interactions on log10(CRP).

Analysis of Age x coinfection interaction on log_10_ [CRP] were statistically significant after FDR adjustment for adenovirus (*q* = 0.049), RSV (*q* = 0.049), common coronavirus (*q* = 0.022), and SARS-Cov-2 (*q* = 0.049), confirming that the relationship between coinfection and CRP varies by age within several pathogens. (Table 5, Figure 1). The pooled fold-change in CRP between coinfection and single virus was largest for parainfluenza (2.5 x, *q* < 0.001), RV/EV (1.47 *q* < 0.001), and RSV (1.40 x, *q* = 0.024).

**Table 5:** Age x coinfection interaction tests on log10(CRP), by virus.

Virus	*n*	Coinf vs. single CRP fold-change	Main effect *p* (MultiVirus)	Age × MultiVirus interaction F	Age × Virus interaction *p*	Main effect *q* (FDR)	Interaction *q* (FDR)
Adenovirus	400	0.87	0.4369	2.833	0.0244	0.4369	0.0494
RSV	689	1.4	0.0088	2.811	0.0247	0.0235	0.0494
Influenza	255	1.3	0.2447	1.259	0.2868	0.3915	0.4589
hMPV	189	0.8	0.3614	0.712	0.585	0.4168	0.6581
Coronavirus	185	1.45	0.1242	4.227	0.0027	0.2484	0.0216
SARS-CoV-2	173	1.31	0.3647	3.052	0.0185	0.4168	0.0494
RV/EV	1,519	1.47	0.0001	0.606	0.6581	0.0004	0.6581
Parainfluenza	265	2.52	0.0001	0.766	0.5482	0.0004	0.6581

Fold-change is 10^beta for the multi-virus indicator from a reduced log10(CRP) model. q = FDR-adjusted *p*-value.

### RSV-specific age-stratified analysis

Among RSV-positive encounters (*n* = 689), CRP was non-significantly higher in coinfection compared with single-virus encounter in children < 6 months of age (5.0 vs. 3.3 mg/L, *p* = 0.07) and 6–23 months (14.4 vs. 8.4 mg/L, *p* = 0.08), but reversed direction in children 2–5 years (15.5 vs. 19.6, *p* = 0.31), and reached nominal significance in 6–12 years (103.4 vs. 11.9, *p* = 0.03), although the latter rests on only 20 encounters. The 13 years and older stratum had insufficient data for analysis (Table 6).

**Table 6:** CRP in RSV-positive encounters, stratified by age group.

Age group	Single *n*	Single CRP median [IQR]	Coinf *n*	Coinf CRP median [IQR]	*p*	Note
<6 mo	164	3.3 [0.8–10.0]	75	5.0 [1.4–18.5]	0.0718	
6–23 mo	136	8.4 [4.2–27.3]	114	14.4 [4.7–37.4]	0.0754	
2–5 y	110	19.6 [5.8–62.4]	62	15.5 [5.2–32.0]	0.309	
6–12 y	13	11.9 [0.5–26.4]	7	103.4 [46.6–173.5]	0.0323	
13–21 y	6	nan	2	nan	nan	Insufficient *n* = *n* < 5

Mann–Whitney U test. Strata with fewer than 5 encounters per group were not tested. na*n* = no additional notes. Blank note fields indicate no additional note; strata with insufficient sample size (*n* < 5) were not tested.

### CRP and hospital disposition

When we stratified by disposition, the difference in CRP between multi-virus and single virus encounters was confined to ED-only patients (single 6.9 vs. coinfection 15.5 mg/L, *p* = 0.008), and was non-significant among children who received care on the pediatric ward (16.3 vs. 14.2, mg/L, p 0.30) or received PICU-level care (8.7 vs. 8.6 mg/L, *p* = 0.58). These observations are consistent with a ceiling effect among hospitalized patients, in whom CRP was broadly elevated regardless of coinfection status (Table 7).

**Table 7:** CRP by coinfection status, stratified by hospital disposition.

Disposition	Single *n*	Single CRP median [IQR]	Coinf *n*	Coinf CRP median [IQR]	*p*
ED only	660	6.9 [1.9–26.1]	82	15.5 [4.6–33.9]	0.008
Ward	1,753	16.3 [3.3–52.9]	220	14.2 [6.2–46.1]	0.3046
PICU	1,883	8.7 [1.8–37.1]	348	8.6 [3.2–31.0]	0.5824

Mann–Whitney U test on CRP within each disposition stratum.

### CRP and advanced respiratory support

In multivariable logistic regression analysis for advanced respiratory support, multicollinearity among candidate covariates was minimal (all VIF < 1.20). In the parsimonious primary model (Model C), each 10 mg/L rise in CRP was associated with an aOR of 1.013 (95% CI 1.001–1.026, *p* = 0.036) for advanced respiratory support; multi-virus aOR 1.27 (1.05–1.54), RV/EV aOR 1.37 (1.18–1.58), preterm birth aOR 1.69 (1.42–2.00) and each percentage-point lower SpO2 aOR 0.83 (0.83 (0.82–0.85). When severity of illness was included as an additional adjuster (Model D), severity dominated the model with an implausibly large aOR of 17.8); we therefore retained the parsimonious specifications as primary and report the Model D for transparency only, noting that severity is not independent of advanced respiratory support (Table 8).

**Table 8:** Multivariable logistic regression for advanced respiratory support.

Model	Variable	aOR	95% CI	*p*
A: Clinical only	AgeYears	0.91	[0.896, 0.925]	<0.001
A: Clinical only	Female	0.796	[0.698, 0.908]	0.0007
A: Clinical only	Preterm	1.67	[1.408, 1.98]	<0.001
A: Clinical only	SpO2	0.826	[0.811, 0.842]	<0.001
B: + CRP	Age Years	0.906	[0.891, 0.922]	<0.001
B: + CRP	Female	0.795	[0.696, 0.906]	0.0006
B: + CRP	Preterm	1.683	[1.418, 1.996]	<0.001
B: + CRP	SpO2	0.827	[0.811, 0.842]	<0.001
B: + CRP	CRP_per10	1.012	[0.999, 1.024]	0.064
C: + Virology	Age Years	0.911	[0.896, 0.927]	<0.001
C: + Virology	Female	0.799	[0.7, 0.912]	0.0009
C: + Virology	Preterm	1.688	[1.421, 2.004]	<0.001
C: + Virology	SpO2	0.832	[0.817, 0.847]	<0.001
C: + Virology	CRP_per10	1.013	[1.001, 1.026]	0.0363
C: + Virology	MultiVirus	1.274	[1.054, 1.54]	0.0122
C: + Virology	RVEV	1.367	[1.184, 1.579]	<0.001
D: + Severity (overadj.)	Age Years	0.908	[0.89, 0.925]	<0.001
D: + Severity (overadj.)	Female	0.889	[0.755, 1.048]	0.1617
D: + Severity (overadj.)	Preterm	1.602	[1.291, 1.989]	<0.001
D: + Severity (overadj.)	SpO2	0.867	[0.849, 0.885]	<0.001
D: + Severity (overadj.)	Severity	17.755	[14.804, 21.295]	<0.001
D: + Severity (overadj.)	CRP_per10	1.028	[1.012, 1.043]	0.0004
D: + Severity (overadj.)	MultiVirus	1.399	[1.096, 1.784]	0.0069
D: + Severity (overadj.)	RVEV	1.134	[0.947, 1.357]	0.1712

Model A: clinical only; Model B: + CRP; Model C (primary, parsimonious): + virology features; Model D: + severity-of-illness, presented for transparency only and known to be over adjusted because severity is partly defined by respiratory support. CRP scaled per 10 mg/L.

When we re-fit parsimonious multivariable model for advanced respiratory support using three additional specifications: (1) logistic regression with cluster-robust (“sandwich”) standard errors on randomized patient identifier; (2) generalized estimating equations (GEE) with binomial family, logit link, independence working correlation, and robust standard errors clustered on patient; and (3) a non-parametric cluster bootstrap, with 95% CI derived from the 2.5–97.5 percentile of the resampled coefficient distribution, the results were similar.

### Discrimination and threshold performance

**Figure 2:**
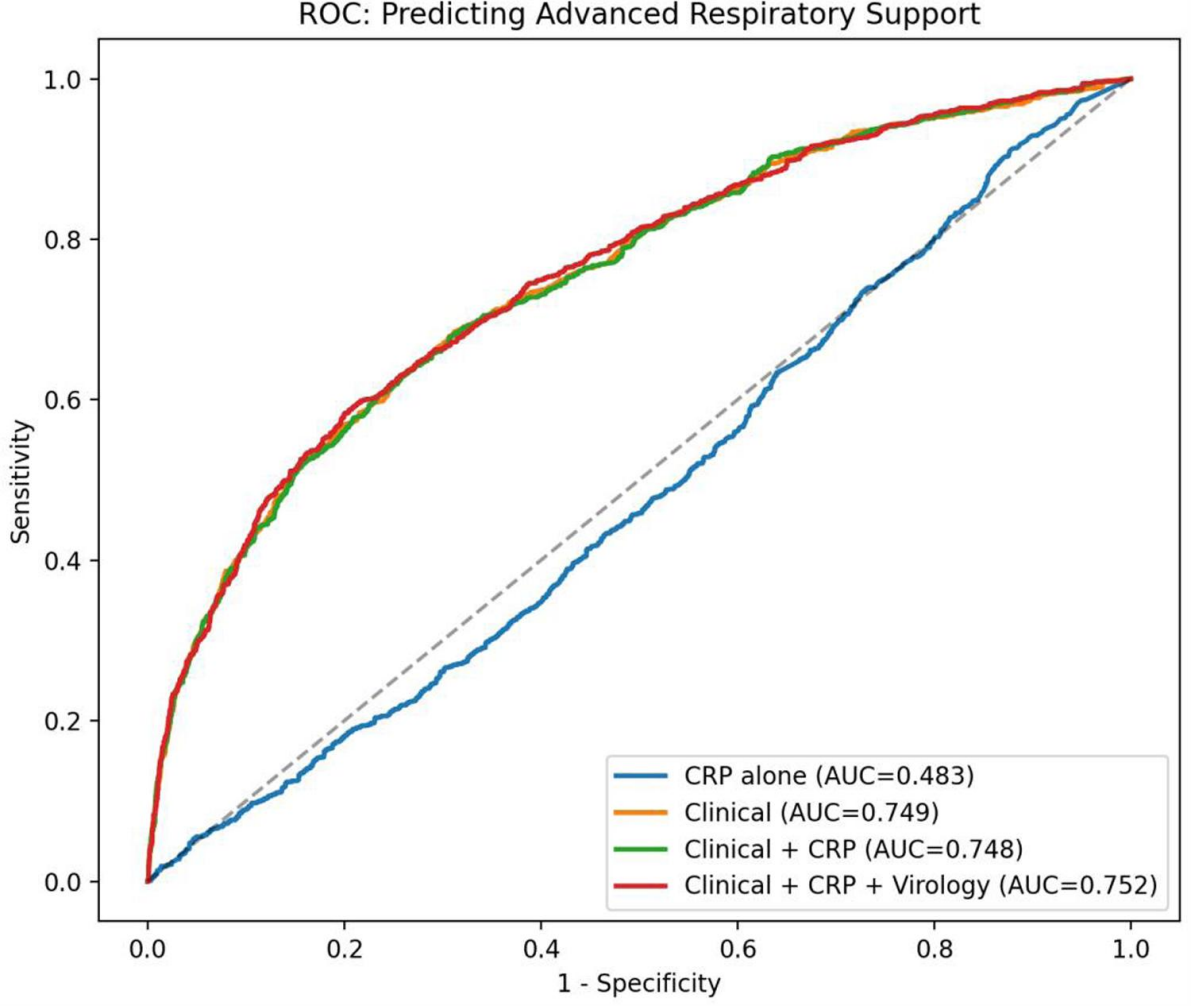
ROC curves for advanced respiratory support during the encounter. CRP alone shows no discrimination (AUC 0.483). A clinical model (age, sex, prematurity, initial SpO2) achieves AUC 0.749. Adding CRP and virology features yields AUC 0.748 and 0.752, respectively.

CRP alone did not show discrimination for advanced respiratory support (AUC 0.48, 95% CI 0.46–0.49). A clinical model containing age, sex, history of preterm birth, and initial SpO_2_ yielded AUC 0.74 (0.73–0.76). Adding CRP produced no improvement (AUC 0.74), adding virology features, improved AUC marginally to 0.75 (Table 9, Figure 2). At conventional CRP cut-offs, sensitivity was uniformly low, and the positive likelihood ratio did not exceed 1.0 at any threshold examined: ≥ 10 mg/L sensitivity 0.48/ specificity 0.48/ + LR 0.92; ≥ 40 mg/L sensitivity 0.22/specificity 0.73/+LR 0.84 (Table 10).

**Table 9:** Discrimination (AUC) for advanced respiratory support.

Model	AUC	95% CI
CRP alone	0.483	[0.466, 0.499]
Clinical (Age, Sex, Preterm, SpO2)	0.749	[0.734, 0.764]
Clinical + CRP	0.748	[0.733, 0.764]
Clinical + CRP + Virology	0.752	[0.737, 0.767]

95% CIs from 1,000-resample non-parametric bootstrap.

**Table 10:** Performance of CRP thresholds for predicting advanced respiratory support.

CRP threshold (mg/L)	*n* above	Sensitivity	Specificity	PPV	NPV	+LR
≥10	2,518	0.482	0.476	0.306	0.657	0.92
≥20	1,919	0.355	0.595	0.295	0.658	0.88
≥40	1,250	0.224	0.733	0.286	0.663	0.84
≥80	676	0.124	0.857	0.293	0.671	0.86
≥100	528	0.098	0.889	0.297	0.673	0.88

PPV, positive predictive value; NPV, negative predictive value; +LR, positive likelihood ratio.

### Robustness to repeated encounters

Of the total of 4,946 CRP-tested encounters, 1,751 represented repeated encounters from 1,132 unique patient. Restricting to one encounter per patient (*n* = 3,195), the multi-virus vs. single-virus difference in CRP attenuated to non-significant (median 9.7 vs. 10.8 mg/L, *p* = 0.08), and the CRP per 10 mg/L coefficient in Model C dropped to aOR 1.00 (0.98–1.018, *p* = 0.96), while multi-virus (aOR 1.24) and RV/EV (aOR 1.45) remained associated with advanced respiratory support (Table 11). The originally observed CRP effect size therefore appears to derive in part from repeated measurement of the same patients.

**Table 11:** Sensitivity analysis: parsimonious logistic model restricted to one encounter per unique patient.

Model	Variable	aOR	95% CI	*p*
First encounter only (Model C)	AgeYears	0.874	[0.853, 0.896]	<0.001
First encounter only (Model C)	Female	0.834	[0.706, 0.986]	0.0339
First encounter only (Model C)	Preterm	1.419	[1.129, 1.782]	0.0027
First encounter only (Model C)	SpO2	0.83	[0.811, 0.849]	<0.001
First encounter only (Model C)	CRP_per10	1.0	[0.983, 1.018]	0.9588
First encounter only (Model C)	MultiVirus	1.337	[1.06, 1.686]	0.0142
First encounter only (Model C)	RVEV	1.445	[1.207, 1.731]	0.0001

First encounter selected as the earliest by admission date/time per randomized MRN.

## Discussion

In this retrospective single center cohort of 17,089 ED encounters with PCR-confirmed pediatric acute respiratory tract infection, and 4,946 encounters with measured CRP spanning 2019 through 2025, we found that the discriminatory value of CRP for viral coinfection and for short term respiratory severity is limited and more context-dependent than commonly assumed (7, 10, 11).

Our results demonstrate that CRP was not measured at random by the treating physician. Patients in the CRP-tested cohort were significantly more likely to be hospitalized, to receive antibiotic in the emergency department, and to be diagnosed with radiographic pneumonia and sepsis than patients in whom CRP was not obtained, with SMD ranging from 0.32 to 0.86. Additionally, RV/EV-Positivity was meaningfully lower among CRP-tested encounters. The CRP analytic cohort is therefore best understood as a high-acuity, a suspected bacterial infection subset of children rather than a representative sample of pediatric viral ARTI (4–6). These findings are consistent with prior studies that demonstrated that inflammatory biomarkers are preferentially obtained in children with perceived illness severity or when clinicians have concerns for bacterial infection (4–6).

When we restricted analysis to encounters without surrogate evidence of bacterial coinfection, most virus-specific differences in CRP between single-virus and multi-virus encounters attenuated and lost statistical significance. The strongest signal was noted in RSV. For this virus in both the full CRP cohort and the viral-only cohort RSV encounter with another virus detected had higher CRP values than RSV-only encounters. Importantly, these results persisted even after excluding encounters with radiographic pneumonia, sepsis, or antibiotic administration. That suggests the higher CRP in RSV co-detection is not entirely explained by obvious bacterial-risk features or antibiotic treatment decisions. It may reflect greater inflammatory burden, more severe viral illness, or the biologic complexity of co-detection. The RV/EV coinfection effect on CRP disappeared and the apparernt adenovirus pattern reversed. These observations together with a formal age x virus interaction analysis, argue strongly against any uniform inflammatory signature of viral coinfection. Systematic reviews evaluating pediatric respiratory viral coinfection have failed to demonstrate a consistent relationship between coinfection and disease severity across pathogens (11, 12).

The parainfluenza finding is also interesting but less robust. In the full CRP cohort, parainfluenza co-detection was associated with higher CRP, but this signal weakened in the viral-only cohort that the parainfluenza finding may be partly driven by encounters with pneumonia, sepsis, antibiotic use, or greater illness severity.

For adenovirus, the direction was different. Single adenovirus detections tended to have higher CRP than adenovirus co-detections. That is biologically plausible because adenovirus can generate a strong inflammatory response on its own, but the FDR-adjusted result was not clearly significant, so we would avoid overemphasizing it.

### For RV/EV, the co-detection effect was weak and not robust after FDR correction

Additionally, the predictive contribution of CRP to short- term respiratory severity was small. CRP alone did not discriminate patients who received advanced respiratory support from those who did not (AUC 0.48). A simple clinical model containing age, sex, preterm birth, and initial SpO_2_ achieved an AUC of 0.74, and adding CRP to this model produced no meaningful improvement. Although CRP retained statistical significance in the parsimonious multivariable model, the effect size (aOR 1.013 per 10 mg/L) was too small to be clinically actionable and disappeared in the one-encounter-per-patient sensitivity analysis. Prior pediatric studies evaluating CRP in bronchiolitis and pneumonia have similarly questioned whether CRP meaningfully improves predication, once bedside clinical assessment is accounted for (3, 5, 13, 14).

An important observation of this study is that the inflammatory signal associated with coinfection was concentrated among milder ED-only encounters and disappeared among children admitted to pediatric wards and PICU, suggesting a potential ceiling effect in severe illness states where CRP becomes broadly elevated regardless of pathogen composition (6, 13–21).

Our results converge with prior systematic reviews and meta-analysis that have failed to demonstrate a consistent association between viral coinfection and disease severity in children with ARTI (11, 12, 17), and they extend that work to inflammatory biomarker interpretation. Methodologically, our findings illustrate the importance of characterizing the population in whom a biomarker is measured rather than assuming representativeness, separating viral-only from probable bacterial encounters before drawing conclusions about virus-specific inflammation, and thus avoiding overadjustment in multivariable models that contain covariates that partially define the outcome itself (8, 9, 17, 18). Finally our repeated-encounter sensitivity analyses suggest that failure to account for within-patient correlation may inflate apparent biomarker association in retrospective respiratory cohorts (22).

### Limitations

As a single center retrospective cohort our findings may not be generalizable to other clinical settings. We did not have access to the full childhood immunization records including influenza immunization records, procalcitonin, viral cycle threshold values, or symptom duration. We used the clinically pragmatic but imperfect surrogate (pneumonia, sepsis, and treatment with an antibiotic) for probable bacterial coinfection. Multiplex respiratory PCR cannot distinguish active replication from prolonged viral shedding, limiting causal inference about coinfection. Our outcome (advanced respiratory support during encounter) reflects acute care intensity that may be subject to local practice variation.

### What are the implications of the main findings in this study?

Collectively, the results of this study suggest that CRP in pediatric ARTI is skewed by who gets tested and how the data are interpreted, an insight that is useful if it is translated into a deployable stewardship tool. To fulfill these expectations, we have introduced the CRP-ARTI-ED rule that is designed to be Artificial Intelligence (AI)-deployable. In this rule each branch is encoded as fields such as age, prematurity, respiratory rate, SpO_2_, respiratory PCR results, including RSV status, and antibiotic-order intent, making it directly compatible with the best-practice-alert (BPA) infrastructure and with emerging large-language-model ED-triage assistants. A CRP recommendation generated by this tool can surface at the point of physician order entry, and either suppresses a low-yield CRP order or promotes one in the narrow RSV-under-2-year subgroup where our data show residual prognostic signal. When we retrospectively applied this rule to our 17,089-encounter cohort, this approach showed that 3,443 of 4,946 (69.9%) of currently ordered CRP tests would have been avoided, while 1,735 selectively targeted orders, would have been added, including 302 in RSV-positive children younger than 2 years. Prospective, multicenter implementation of CRP-ARTI-ED, embedded as either BPA or an LLM triage output, or both, is the logical next step, and falls beyond the scope of the present analysis.

## Conclusions

In this 17,089-encounter pediatric ARTI, the largest single-center to date, jointly analyzing CRP, multi-pathogen PCR, and advanced respiratory support outcome, clinicians preferentially performed CRP testing in children who were more seriously ill or were suspected to have bacterial coinfection. After accounting for this indication bias, repeated encounters, probability of bacterial coinfection, and overadjustment, CRP did not reliably distinguish single-from multi-virus infection – apart from a modest signal in RSV-positive children and CRP did not provide clinically useful incremental discrimination for advanced respiratory support measures beyond age, sex, prematurity, and initial SpO_2_, These findings can potentially reframe CRP from a presumed prognostic biomarker into a laboratory test whose apparent value largely reflects who gets tested in this clinical setting and how the data are analyzed. Clinicians should rely on patient clinical data, rather than use CRP values as a standalone trigger for escalation of respiratory support. CRP has a limited value as a surrogate for viral coinfection burden; its prognostic value is small, age- and pathogen dependent, and cannot replace clinical assessment.

## Data Availability

The datasets presented in this article are not readily available because the institution limits data to be used within the institution only. Requests to access the datasets should be directed to tmiller13@hurleymc.com.
